# Survivorship and risk factors for revision after total hip arthroplasty in patients 30 years and younger: a cohort study from the German arthroplasty register

**DOI:** 10.2340/17453674.2025.45042

**Published:** 2026-01-03

**Authors:** Johanna ELLIOTT, Yinan WU, Arnd STEINBRÜCK, Alexander W GRIMBERG

**Affiliations:** 1 German Arthroplasty Registry (EPRD Deutsche Endoprothesenregister gGmbH), Berlin, Germany; 2 University of Newcastle, Hunter New England Health District, NSW, Australia; 3 Department of Orthopaedic Surgery, Physical Medicine and Rehabilitation, University Hospital of Munich (LMU), Munich, Germany

## Abstract

**Background and purpose:**

Total hip arthroplasty (THA) in young patients is rare but increasingly performed. We aimed to analyze implant survivorship and risk factors for revision in patients aged 30 years or less after THA based on the German Arthroplasty Registry (EPRD).

**Methods:**

Kaplan–Meier survival analysis and Cox proportional hazard models were used to analyze the EPRD dataset from 2013 to 2023 for factors associated with increased risk of revision. The primary outcome was first revision operation.

**Results:**

1,622 primary THAs in 1,452 patients were analyzed. The mean age was 26 years (range 11–30), 908 (56%) of whom were male. The most frequent diagnosis was secondary osteoarthritis (1,146, 72%), followed by osteonecrosis (357, 22%), and hip dysplasia (53, 3.2%). Of all THAs, 1,601 (99%) were uncemented, and 1,574 (97%) received ceramic heads. The average follow-up period was 3.7 years (range 0–10.6). 47 hips were revised with a cumulative revision rate (CRR) at 8 years of 4.6% (95% confidence interval [CI] 2.8–7.3). The most frequent revision cause was infection in 11 cases (0.7%). Increased revision risk was associated with pediatric hip disease for those with prior surgery for Perthes, HR 4.3 (CI 1.9–9.6), pelvic osteotomy HR 2.8 (CI 1.1–7.5), and a primary diagnosis of hip dysplasia, HR 3.4 (CI 1.3–8.5).

**Conclusion:**

Uncemented THA in young patients demonstrated a revision rate of 4.6% (CI 2.8–7.3), which we believe is a satisfactory mid-term survival. Patients with pediatric hip disease present the highest risk of revision.

Total hip arthroplasty (THA) [1] is the last option for end-stage hip osteoarthritis (OA) in younger patients [2]. It is postponed as long as possible as higher activity levels observed amongst these patients [3] may predispose to implant loosening and early revision [4].

The pathogenesis of end-stage hip pathology in patients aged 30 years or less differs significantly from that of the typical THA cohort [5-7]. Unlike idiopathic degenerative OA of the hip affecting the elderly population, congenital, metabolic, inflammatory, and neoplastic pathologies contribute more frequently to end-stage hip joint pathology in juveniles [2,8].

Improved quality of life is reported by young patients undergoing THA [5]. Advances in materials, fixation techniques, and tribology have coincided with improved survivorship from childhood hip disease, contributing to an expansion of the indication for THA in younger age groups [8,9]. This is reflected in the stable projected THA incidence rate reported for those aged 45 or less in Germany [10], despite reticence from those indicating the surgery.

Because of the scarcity of THA in young populations, previously published registry studies describe implant survivorship in cohorts over several decades [5,8,11-13]. Over these long observation periods, implants, fixation methods, and tribology vary significantly, such that the time frame during which the surgery was performed becomes an independent factor influencing revision risk [5,11,12,14]. The advantage of the German Arthroplasty Registry (EPRD) database, one of the largest in Europe [15], is that the substantial size of the age-defined cohort undergoing a comparatively uniform procedure within a single decade minimizes the impact of surgical trends seen to vary over decades.

The aim of our study was to analyze the EPRD database to determine implant survivorship and risk factors for revision in patients aged 30 years or less after THA.

## Methods

### Study design

This population-based observational study utilized the EPRD database on THAs. All primary THAs performed on patients aged 30 years or less at the index operation were included between 2012 and 2023. Those receiving metal-on-metal (M-o-M) articulations were excluded, and those operations where the primary indication for the surgery was missing were also excluded. Data on patient covariates, implant specifics, and technical influences was collected, as well as revision rate and reason for revision. Frequency tables describe population characteristics and implant specifics. Analyses were conducted to determine implant survivorship and covariates associated with revision.

The study is reported according to STROBE guidelines.

### Data source

The EPRD started collecting data in November 2012 and is a not-for-profit organization founded by surgeons and the German Society of Orthopaedics and Orthopaedic Surgery (DGOOC) in cooperation with public health insurers (AOK Bundesverband GbR, Verband der Ersatzkassen e.V, vdek), the German Medical Technology Association (BVMed), and hospitals performing hip and knee arthroplasty. Voluntary inclusion in the national registry database currently stands at approximately 78% of operations reported in 2023, correlating numbers of EPRD with those of the German Institute for Quality Assurance and Transparency in Healthcare (IQTIG). The 2 participating insurance companies (AOK-B, vdek) cover around 65% of the German population and allow cross-validation with patient healthcare data. Because of the linkage between health insurance and EPRD data, loss of follow-up revision surgery is minimized for patients meeting the inclusion criteria.

Cross-validation process ensures high data quality with a closed system for completeness of revisions. The EPRD uses the German version of the International Classification of Procedures in Medicine (ICPM), known as the “Operationen und Prozedurenschlüssel” (OPS) 301 system, and the 10th International Classification of Diseases (ICD-10) to classify and identify diagnoses and procedures accurately. Where a primary arthroplasty is revised in a German hospital, the EPRD relies on the German “Medizinischer Dienst der Krankenversicherung” (MDK) (medical review service for statutory health insurance funds) whose task it is to correlate hospital billing data with coded diagnoses and procedures ensuring that they are medically justified, correctly documented, and compliant with coding and reimbursement rules. Accounting verification is provided even if this hospital does not routinely provide data to the EPRD. Only cases that are revised and billed outside of Germany escape inclusion in the closed system. The few such cases that most likely exist remain unaccountable. Similarly, revision cases flagged as “infection” to the EPRD are verified via the electronic case report form (eCRF) or by the MDK when reimbursement data is coded ICD-10 T84.5, “Infection and inflammatory reaction due to internal joint prosthesis.”

### Study population

No THAs were performed in patients aged 30 years or less in the EPRD database in 2012. The EPRD currently has over 570,000 primary THAs performed between 2013 and 2023 under surveillance.

### Covariates

Information on patient age at index operation, sex, BMI, ASA, Elixhauser Score, primary diagnosis as coded in the OPS system, calendar year of surgery, documented prior “relevant” surgery on the hip, type of fixation, implants, grade of acetabular component complexity, articulations, femoral head size, cause of any revision, and month of death were collected.

Primary diagnoses coded as M16.0 and M16.1 (primary osteoarthritis [OA], bilateral or unilateral) were considered as idiopathic primary OA, while those coded M16.6, M16.7, or M16.9 were categorized for the purpose of this analysis as secondary OA. Other categories of specific forms of OA included osteonecrosis of the femoral head, hip dysplasia, inflammatory arthropathy, post-traumatic, and other.

Documentation of “relevant prior hip surgery” is recorded in the ERPD at the time of arthroplasty as either no prior relevant surgery, surgery of the pelvis, femur, femur and pelvis, surgery for femoral head necrosis, and other. The category of other may include, but is not limited to, soft tissue and arthroscopic surgery. The nature of prior relevant surgery for the purposes of this study was not verified against the patient medical record.

### Outcome measures

The primary endpoint was time to first revision. Revision was defined as any removal or exchange of any component including liner exchange.

Data on reason for revision was categorized as dislocation, infection, loosening acetabular/acetabular and femoral/femoral, malalignment, missing, osteolysis, periprosthetic fracture, and other.

### Statistics

Descriptive data of the population is summarized in a frequency table (see Table 1).

**Table 1:** Frequency of patient characteristics (N = 1,622)

Factor	n (%)
Age, years	
< 20	184 (11)
≥ 20	1,438 (89)
Sex	
Male	908 (56)
Female	714 (44)
Body mass index	
< 30	946 (58)
≥ 30	256 (16)
Missing	420 (26)
ASA class	
1	259 (16)
2	373 (23)
3	87 (5.3)
4	2 (0.1)
Missing	901 (56)
Vital status – dead	15 (0.9)
Primary diagnosis at index operation	
Osteoarthritis, primary	20 (1.2)
Osteoarthritis, secondary	1,146 (71)
Osteonecrosis	357 (22)
Hip dysplasia	53 (3.3)
Inflammatory arthritis	23 (1.4)
Trauma	12 (0.7)
Other	11 (0.7)
Previous surgery	
None	1,159 (72)
Pelvis	56 (3.5)
Femur	163 (10)
Pelvis and femur	47 (2.9)
Femoral head necrosis	77 (4.7)
Other	120 (7.4)
Year of operation	
< 2020	842 (52)
≥ 2020	780 (48)

ASA = American Society of Anesthesiologists Physical Status classification.

Continuous data was described using means and standard deviation (SD). For the purposes of analysis, continuous data (age at operation, BMI, era of surgery) was binarized and treated as categorical. Age at time of surgery was binarized into those aged less than 20 at the time of surgery, and those 20 years and older. Data on BMI was binarized as less than 30 and 30 or greater. Era of operation was binarized as surgery conducted between 2012 and 2020, and from 2020 to the end of the study period. The diagnoses for primary THA were grouped into 6 categories: secondary OA, osteonecrosis, hip dysplasia, inflammatory arthropathy, trauma, and other. Categorical variables were summarized as frequencies and percentages.

Patient death during the observation period was tabulated as “vital status.” With the death of the patient, observational follow-up is ceased and the case “censored” from implant follow-up, avoiding underestimation of the revision rate.

The categorical data on choice of implants used is depicted in a frequency table (see Table 2).

**Table 2:** Frequency of implant characteristics (N = 1,622)

Factor	n (%)	n (%)
Acetabular fixation ^**a**^		
Cemented		14 (0.9)
Uncemented		1,607 (99)
Femoral fixation ^**a**^		
Cemented		10 (0.6)
Uncemented		1,611 (99)
Acetabular bearing ^**a**^		
Polyethylene		1,153 (71)
Ceramic		468 (29)
Acetabular component complexity		
Simple	1,524 (94)	
Monobloc		67 (4.1)
Modular		1,457 (90)
Complex	94 (6.0)	
Dual mobility		17 (1.0)
Revision		77 (4.7)
Unknown		4 (<0.1)
Femoral bearing ^**a**^		
Metal		47 (2.9)
Ceramic		1,574 (97)
Head size, mm		
< 32	257 (16)	
22		22 (1.4)
22.25		14 (0.9)
28		221 (14)
≥ 32	1,365 (84)	
32		794 (49)
36		569 (35)
40		1 (<0.1)
44		1 (<0.1)

a 1 missing data point for implant characteristics.

Factors influencing surgical choice for cemented components are described in a frequency table using frequencies and percentages (see Table 3).

**Table 3:** Characteristics of patients receiving cemented implants. Values are count/total number available and (percentage)

Factor	Cementless n = 1,612	Cemented n = 10
ASA class		
1	258 / 716 (36)	1 / 5
2	373 / 716 (52)	0 / 5
3	84 / 716 (12)	3 / 5
4	1 / 716 (0.1)	1 / 5
Vital status – dead	13 / 1,612 (0.8)	2 / 10
Year of surgery		
< 2020	837 / 1,612 (52)	5 / 10
≥ 2020	775 / 1,612 (48)	5 / 10

For the purposes of analysis and clinical relevance, the reasons given for revision were re-grouped into 5 categories: infection, dislocation, aseptic loosening (acetabular femoral, or both components), periprosthetic fracture, and other (progression of arthrosis, osteolysis, malalignment, missing).

Statistical analysis was performed using R statistical software (Version R-4.4.0., R. Foundation for Statistical Computing, Vienna, Austria). A P value threshold of 0.05 was considered indicative of statistical significance.

The Kaplan–Meier method was used to calculate unadjusted survival functions. Univariate Cox regressions were used to calculate the hazard ratio (HR) and accompanying 95% confidence interval (CI) for each covariate.

### Ethics, funding, and disclosures

This study was approved by the ethics committee of the medical school of Kiel University (approval number D 473/11). The corresponding author received funding for presentation of the initial results of this study at the ISAR Congress 2025. No conflict of interest was reported by the authors. Complete disclosure of interest forms according to ICMJE are available on the article page, doi: 10.2340/17453674.2025.45042

## Results

**Figure 1:**
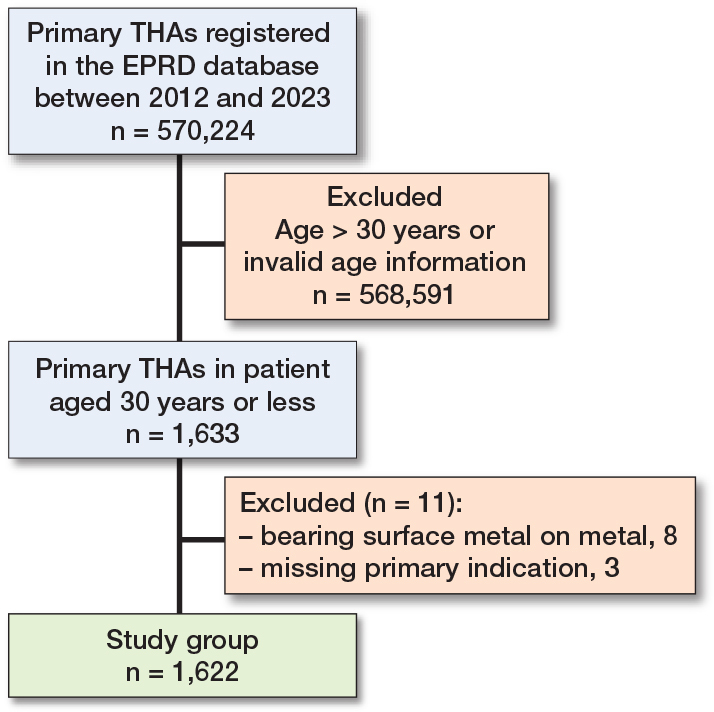
Flowchart of the inclusion process.

We included 1,460 patients with a documented primary diagnosis receiving THAs. 8 hips (0.5%) receiving a M-o-M articulation were excluded from analysis, leaving 1,622 THAs in 1,452 patients, 170 (11%) of whom underwent bilateral THA (Figure 1).

Approximately half (842, 52%) of the cohort were operated on in the 8 years prior to 2020, and the remainder in the 3 years from 2020 (Table 1).

In this cohort, THA was more frequently performed in males (908, 56%) than females. Mean age at primary THA was 26 (range 11–30) years (SD 4.2). Secondary OA was most frequently given as the indication for THA in 1,165 hips (72%). Pediatric hip disease including hip dysplasia and osteonecrosis of the femoral head were the next most frequently observed categories, followed by inflammatory arthritis, trauma, and other.

Of the 463 hips (29%) undergoing prior surgery, the category most frequently nominated was “femur” in 163 (10%) cases, followed by “other” in 120 (7.4%) cases.

### Prosthetic concept, articulation, and head size

Of the 1,622 THAs, 1,601 (99%) were uncemented, 4 were fully cemented, and 6 hybrid; 10 reverse hybrid arthroplasties were documented and 1 data point was missing (Table 2).

Simple “modular” or “monobloc” acetabular components were implanted in 1,524 (94%) of cases. A femoral head size of at least 32 mm was implanted in 1,365 (84%) hips and 1,574 had ceramic femoral heads. On the acetabular side, the bearing surface was highly cross-linked polyethylene in 1,153 cases (71%) and ceramic in the remaining 468 cases (29%).

4 of the hybrid THAs were assigned to patients with ASA grade 3 or higher. Patients receiving cemented components had higher comorbidity status as indicated by the higher Elixhauser score and reduced life expectancy as documented by vital status (alive or dead) (Table 3).

### Implant survivorship and reasons for revision

**Figure 2:**
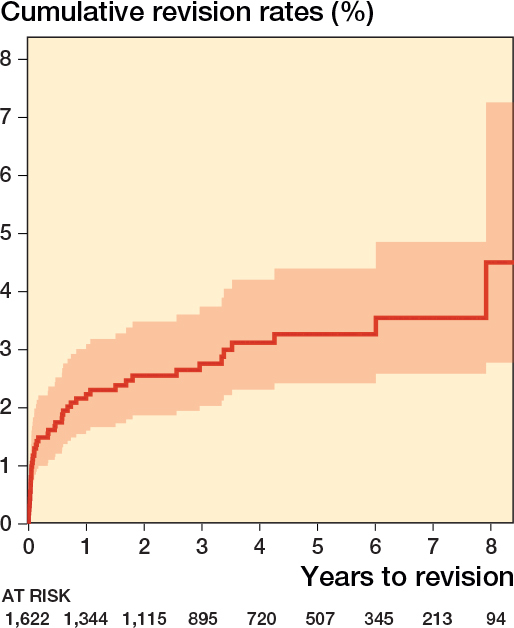
Kaplan–Meier cumulative revision rates.

47 revisions were observed. At 8 years, the cumulative revision rate (CRR) was 4.6% (CI 2.8–7.3). Kaplan–-Meier analysis showed a flattening of the revision curve after failures within the first 12 months (Figure 2).

Most revisions were performed due to infection, followed by aseptic loosening and dislocation (Table 4). In 19 cases, infection was excluded but no reason for revision was specified. There was no statistically significant association between the clinical and implant covariates, or the reason given for revision.

**Table 4:** Reason for revision, n = 47

Reason	n
Aseptic loosening	
Acetabulum	5
Femur	3
Both	1
Infection	11
Dislocation	5
Periprosthetic fracture	2
Other (osteolysis, malalignment, missing)	20

### Factors associated with revision

We showed an increased HR of 3.4 (CI 1.3–8.5, P = 0.01) in patients with hip dysplasia (Table 5). The numbers per primary diagnosis in other categories were at times too small to make meaningful estimates of revision risk related to disease.

**Table 5:** Univariate analysis of patient, implant, and technical factors associated with increased risk of revision THA

Factor	HR (CI)	P value
Prior hip surgery (ref. none)		
Pelvis	3.3 (1.3–8.5)	0.01
Femur	0.22 (0.03–1.6)	0.1
Pelvis and femur	0.72 (0.10–5.3)	0.7
Femoral head necrosis	4.0 (1.9–8.8)	< 0.001
Other	0.31 (0.04–2.2)	0.2
Sex (ref. female)		
Male	0.67 (0.38–1.2)	0.2
Age at THA (ref. < 20 years)		
≥ 20 years	0.59 (0.28–1.3)	0.2
Body mass index (ref. < 30)		
≥ 30	1.5 (0.68–3.2)	0.3
Missing	1.1 (0.56–2.2)	0.8
Year of surgery (ref. < 2020)		
≥ 2020	1.3 (0.72–2.5)	0.4
Articulation (ref. ceramic/PE)		
Ceramic/ceramic	0.99 (0.52–1.9)	> 0.9
Metal/PE	2.2 (0.68–7.3)	0.2
Missing	0	
Head size (ref. ≥ 32 mm)		
< 32 mm	2.1 (1.1–4.0)	0.03
Primary diagnosis (ref. secondary OA)		
Osteonecrosis	1.5 (0.75–2.9)	0.3
Hip dysplasia	5.0 (2.1–12)	< 0.001
Inflammatory arthritis	1.7 (0.23–13)	0.6
Trauma	3.9 (0.53–29)	0.2
Other	0	

HR = hazard ratio; CI = 95% confidence interval; OA = osteoarthritis; PE = polyethylene; THA = total hip arthroplasty.

There was no association between sex (male, HR 0.67, CI 0.38–1.2), age (≥ 20 years, HR 0.97, CI 0.90–1.03), the era of surgery (≥ 2020, HR 1.3, CI 0.72–2.5), or BMI (≥ 30, HR 1.5, CI 0.68–3.2) and revision risk.

Prior surgery was identified as a factor associated with an increased risk of revision. Of the 463 hips with prior surgery, 16 (3.5%) were revised. A sub-analysis of the type of surgery revealed an association between specific surgeries for pediatric-related hip pathology with an increased HR of 4.3 (CI 1.9–9.6, P < 0.001) for patients who had undergone surgery for osteonecrosis, and an increased HR of 2.8 (CI 1.1–7.5, P = 0.04) associated with prior surgery on the pelvis.

There was a trend towards increased revision risk with hybrid fixation with 1 of the hybrid cases being revised (HR 5.5, CI 0.75–40, P = 0.09). None of the 4 fully cemented hips were revised. Due to small numbers, an association with cementation did not demonstrate statistical significance.

An increased HR of 2.1 (CI 1.1–4.2, P = 0.03) was detected with a head size smaller than 32 mm.

## Discussion

Our study represents one of the largest contemporaneous arthroplasty registry cohorts in younger patients. Given the implications for young patients, quantification of survivorship and investigation of factors associated with revision is justified.

The aim of our study was to analyze implant survivorship and risk factors for revision in patients aged 30 years or less after THA. We showed a cumulative 8-year revision rate of 4.6%, which we believe is a satisfactory mid-term survival. Patients with pediatric hip disease present the highest risk for revision.

### Survivorship

The findings of this study support those of other registry studies reporting an optimistic outcome of THA in a younger cohort. In 2018, Metcalfe et al. [12] published a Kaplan–Meier estimation of 96% (CI 94–98%) survivorship at 5 years amongst their NJR cohort of patients aged 20 years and less. van Kouswijk et al. [8] published 5- and 10-year cumulative survival rates of 95% (CI 91–97%) and 91% (CI 84–95%) in 2025 on a Dutch Registry cohort of 283 THAs in patients aged 11–18. The median follow-up was 7 years (range 2–16) and 14 revisions were reported. The event of revision was so rare that no statistical definition of a risk factor for revision could be determined for any of the variables analyzed, which included primary diagnosis, patient and implant characteristics, previous surgery, and surgical approach. In contrast to our study, this study was limited to the pediatric population and patients with a primary diagnosis of tumor were excluded.

### Influence of primary diagnosis

In our study, the strongest association with a risk of revision for any reason was pediatric hip disease manifesting as prior surgery for osteonecrosis or pelvic osteotomy, or a primary diagnosis of hip dysplasia. In the literature, however, controversy remains regarding the association between primary diagnosis and the risk of revision THA in young cohorts. Arthroplasty registries collect data on primary diagnosis, but the quality of data can be problematic and misclassification by primary diagnosis can occur. Many studies are not powered to detect differences associated with rarely occurring primary diagnoses and therefore group pediatric hip pathologies including developmental hip dysplasia, slipped capital femoral epiphysis, and Perthes disease together for the purpose of analysis. The New Zealand Registry study by Boyle et al. [16], determined no clinical difference in the outcome of THA amongst all patients with a primary diagnosis of OA, and those with slipped capital femoral epiphysis. While the 2012 Nordic Arthroplasty Registry study [17] combining data from Denmark, Norway, and Sweden, concluded no difference in the overall risk of revision of primary THA after pediatric hip diseases in general compared with primary OA, adjusting for age, sex, and type of fixation of the implants. However, sub-analysis showed an increased risk of revision for dislocation for THA for developmental dysplasia.

Both these registry studies reported on the influence of a primary pediatric hip pathology on THA revision rates in a generic (age-unrestricted) arthroplasty population. However, it is well established that the primary diagnosis in young populations undergoing THA varies significantly from older cohorts [5,18]. Considering THAs restricted to a younger cohort, it is reasonable to consider that these study subjects represent the more severe spectrum of primary pediatric hip pathologies as they become clinically symptomatic at an earlier stage than those subjects included in age-unrestricted registry studies. Girard et al. [1], in their 2011 retrospective review of 77 revisions amongst 941 primary THAs in patients under 30 years, identified generalized “pediatric hip disease” and sequelae from sepsis as associated with a greater risk of revision. In contrast, Hanouche et al. [19] in 2015 reported a 10-year 90.3% survivorship amongst 113 C-o-C-bearing THAs performed over 34 years between 1979 and 2013 in patients younger than 20 and was not powerful enough to identify an association of revision risk with primary diagnosis. Halvorsen et al. [11] analyzed 881 THAs in patients aged 21 years and younger and determined no increased revision risk associated with “pediatric” as primary diagnosis category. Similarly, in 2019 Mohaddes et al. [5] compared THA survivorship of a cohort of 504 THAs implanted over 16 years between 2000 and 2016 with a matched cohort aged over 30, and found no association with “OA following childhood disease.” The findings of our study specify an increased risk of revision associated with both a primary diagnosis of hip dysplasia and prior surgery for pediatric hip conditions. Unlike the findings of Engesaeter et al. [17], we could not establish a statistical association between a primary diagnosis of hip dysplasia and revision for dislocation.

### Mechanism of failure

Previous publications have concluded that causes of revision amongst a juvenile THA cohort differ from those of an older cohort [1,6].

In our study, the most common category nominated was “other,” a category including but not limited to the disparate clinical codes of osteolysis, “missing,” and “other.” The most frequently defined reason for revision was infection, followed by aseptic loosening and dislocation. This result is consistent with the findings of the registry report by Metcalfe et al.[12] on 35 revisions amongst 769 THAs in patients 20 years and younger over 14 years between April 2003 and March 2017 with a median follow-up of 5.1 years where the most frequent reasons given were loosening and infection (both 20%). The authors included M-o-M bearings in their analysis and this was identified as a risk for revision. Similarly to our study, most implants were uncemented and the preferred bearing surface was C-o-C, which reduced the risk of revision.

In our study, infective revisions were more likely to be male, in accordance with published registry data [20]. Due to the relatively high rate of prior hip surgery amongst male patients in this cohort, infection amongst males may be confounded by the event of prior hip surgery.

THAs with smaller head size were at increased risk of revision in keeping with previously published data [21]; however, this was not linked to revision due to dislocation in this cohort. There was a weak trend for increased risk of revision with hybrid fixation.

Mechanism of failure may be influenced by the period in which the study is performed, reflecting the type of implants used. In their retrospective review of 108 revised primary THAs performed between 1982 and 2007 in patients under 35, Kahlenberg et al. [6] identified cup loosening independent of polyethylene wear as the most frequently identified mechanism of failure. In 2011, Girard et al. [1] determined that aseptic loosening associated with hard-on-soft bearings was the most frequent mechanism of failure in their retrospective clinical and radiological review of 77 revisions of THAs performed between 1985 and 2003. As in our study, the authors could not identify an association with cemented fixation. Our study was conducted after widespread introduction of highly crosslinked polyethylene and polyethylene wear (osteolysis) did not feature as cause of revision.

In their Nordic Arthroplasty Registry Association review in 2019, Halvorsen et al. [11] determined no association between cementation and revision risk. Cup revision was more likely than stem revision, and reverse hybrid configurations carried a higher risk of revision.

Mohaddes et al. [5] compared THA survivorship of a cohort of 504 THAs implanted over 16 years between 2000 and 2016 with a cohort aged over 30, determining that in both groups failure for aseptic loosening was the most common reason for revision and cup exchange the most frequently undertaken revision. An increased risk of revision was reported if cemented implants were used.

In Hannouche’s cohort of C-o-C THAs published in 2016 [19], aseptic loosening without ceramic fracture or wear was the most frequent cause of revision.

A strength of this study is that for this cohort of German patients age 30 years or less, observation within the parameters of the EPRD results in highly conclusive data due to linkage and reporting mechanisms [22]. As long as care was provided in a German hospital, the event of infection is validated by the MDK, as is revision surgery for any reason.

### Limitations

There may be misclassification of the diagnosis given, prior hip surgery, and reason for revision. The category of “osteonecrosis” may encompass both pediatric hip disease of Perthes, and adult-type osteonecrosis. Regardless, the frequency with which the category “other” was nominated illustrates the imprecision of this registry data.

Non-infective revisions, suggesting mechanical failure, also lack granularity in data collection.

The methodology of data collection at the EPRD does not include documentation of the exact components revised, only that at least 1 component has been revised. Therefore, infections where no exchange of components occurred, as well as peri-implant fracture managed without revision arthroplasty, are not taken into account in this study.

The EPRD does not routinely collect data on surgical approach, which may impact risk of revision for instability [13,21]. Finally, long term outcome studies of hip arthroplasty in young patients are supplemented by parallel assessment of patient-related outcome measures. The EPRD commenced trialing acquisition of patient-reported outcome measures (PROMs) data in 2023 but is yet to include this in the database routinely.

### Conclusion

Uncemented THA in young patients demonstrated a revision rate of 4.6%, which we believe is a satisfactory mid-term survival. Patients with pediatric hip disease present the highest risk for revision.

*In perspective*, further research is needed to assess long-term outcomes beyond 10 years.

## Notes

JE: conceptualization, formal analysis, funding acquisition, investigation, methodology, project administration, validation, writing—original draft, writing—review and editing. YW: data curation, formal analysis, investigation, methodology, project administration, resources, software, validation, writing—original draft. AS: formal analysis, funding acquisition, investigation, methodology, project administration, resources, supervision, validation, visualization, writing—original draft, writing—review and editing. AWG: funding acquisition, supervision, validation.

Handling co-editors: Marianne Westberg and Robin Christensen

*Acta* thanks Maziar Mohaddes and Lene Bergendal Solberg for help with peer review of this manuscript.
